# Case of a Large Sub-Cutaneous Nævus Cured by Vaccination

**Published:** 1852-05

**Authors:** John Woolcott

**Affiliations:** Surgeon to the Kent Ophthalmic Hospital


From the London Lancet.

*Case of a Large Sub-Cutaneous Nœvus Cured by Vaccination.* By John Woolcott, Esq., M.R.C.S., Surgeon to the Kent Ophthalmic Hospital.

A lady brought to me her infant, a healthy looking child, nine weeks old, in January, 1848, with an extensive sub-cutaneous nævus which had existed from birth. The tumor, which was of a blue, livid color, occupied the whole of the upper eyelid and a small portion of the root of the nose on the right side, and extended upwards upon the brow and forehead as high as the upper border of the orbicularis palpebrarum muscle; outwards and downwards it reached nearly to the tragus of the right ear, and then extended upwards and inwards along the lower margin of the zygomatic process of the temporal bone to the external angle of the orbit, where it joined the morbid product at the upper eyelid; there was no pulsation in the tumor; it was soft and compressible, and increased greatly when the child cried, and it then assumed a dark purple color; pressure on the temporal arteries did not diminish its bulk. The application of ligatures in this case was of course inadmissible, on account of the deformity which would arise from cicatrization of the wound causing ectropium.—The treatment for the first month consisted in the application of tincture of iodine; the abnormal growth being freely punctured all over with a fine cataract needle, and the iodine applied over the punctures. The bleeding was considerable, and of arterial character, but it soon subsided on the application of the iodine. These punctures were made twice a week, but the iodine applied daily, except when it caused too great irritation and soreness of the skin, when it was discontinued for a day or two, and then resumed. At the end of the month, the disease remaining undiminished, I altered the treatment and applied vaccine lymph: with a lancet armed with the matter, punctures were made at short intervals all around the circumference of the tumor, and several points in the centre of it; to ensure its taking, I inserted into each puncture a bone-point, also well-armed with vaccine lymph; most of these punctures took, and the irritation they caused was considerable, the child's face and head being swollen enormously. This was attended with fever and much constitutional disturbance, but at the end of a fortnight it had somewhat abated, and at the end of a month the disease was evidently decreasing; and at the expiration of six months from the vaccine lymph having been used, not the least swelling existed, and the skin was assuming its natural color. I saw the child the beginning of January, 1852, and not a vestige of the morbid structure remains; and it was only by looking closely for the vaccination scars, that I could tell on which side the nævus had been. I have treated several cases in the same way at the Kent Ophthalmic Hospital, and have succeeded in arresting their growth, but I have never seen so large an erectile tumor cured by this treatment, nor can I remember to have read of any such case. The color of this vascular tumor was venous, the bleeding was arterial.

